# Meta-analysis showing that early response to neoadjuvant chemotherapy predicts better survival among cervical cancer patients

**DOI:** 10.18632/oncotarget.19425

**Published:** 2017-07-21

**Authors:** Zhilan Chen, Yachen Shi, Shixuan Wang, Qiping Lu

**Affiliations:** ^1^ Department of Obstetrics and Gynecology, Wuhan General Hospital of Guangzhou Military Command, Wuhan, Hubei, China; ^2^ School of Public Health, Tongji Medical College, Huazhong University of Science and Technology, Wuhan, Hubei, China; ^3^ Department of Obstetrics and Gynecology, Tongji Hospital, Tongji Medical College, Huazhong University of Science and Technology, Wuhan, Hubei, China; ^4^ Department of General Surgery, Wuhan General Hospital of Guangzhou Military Command, Wuhan, Hubei, China

**Keywords:** cervical cancer, early response, survival, neoadjuvant chemotherapy, meta-analysis

## Abstract

This study was designed to identify the prognostic value of early response to neoadjuvant chemotherapy (NACT) for long-term survival of cervical cancer patients. We searched Pubmed and EMBASE for studies published through July 2016 on outcomes of cervical patients that received NACT. Eight studies involving 825 cervical cancer patients were ultimately included in our meta-analysis. We pooled the hazard ratios (HR) according to random-effects models and used funnel plots with Egger's and Begg's tests to explore potential publication bias. The HR between early response and 1-year overall survival (OS) was 3.60 (95% CI 1.93–6.72; *I*^2^ = 0). Similar results were found in the analysis of 3-year OS (HR 3.34; 95% CI 2.28–4.90; *I*^2^ = 0) and 5-year OS (HR 3.44; 95% CI 2.40–4.94; *I*^2^ = 0). Sensitivity analysis showed that all of the pooled results were robust, and all logHRs had confidence limits > 0. Our findings indicate that early response is associated with long-term survival, and responders achieved a higher survival rate than non-responders.

## INTRODUCTION

Cervical cancer is one of the most common cancers in females, second only to breast cancer in developing countries [1]. According to the latest report, there are 527,600 new cases and 265,700 deaths across the world every year [1]; there were 98,900 new cases in China in 2015 [2]. NACT plus surgery, has emerged as a new hope for cervical cancer patients [3–5] and an alternative to traditional therapies, such as radiotherapy or concurrent chemoradiotherapy. First, NACT often reduces tumor size [6], eliminates distant micro-metastasis, and thus makes surgery easier [7–9]. This is particularly important to patients in developing areas [10, 11], where sophisticated radiation equipment and expert radiologists are rare [11]. Second, for patients who cannot endure radiation, NACT largely protects vaginal and ovarian function. As a result, patients enjoy a relatively high quality of life, compared with those who receive traditional radiation therapy [12]. Third, with the help of NACT and radical trachelectomy, young women with cervical cancer can preserve their fertility [12, 13], even for those who are already pregnant at the time of treatment [14].

However, it remains controversial whether early response to NACT is an indicator of long-term survival in patients with cervical cancer, as previous studies provide conflicting results. We designed this study to identify the prognostic role of clinical response on OS by making a pooled analysis of the results from published studies.

## RESULTS

### Literature search

**Figure 1:**
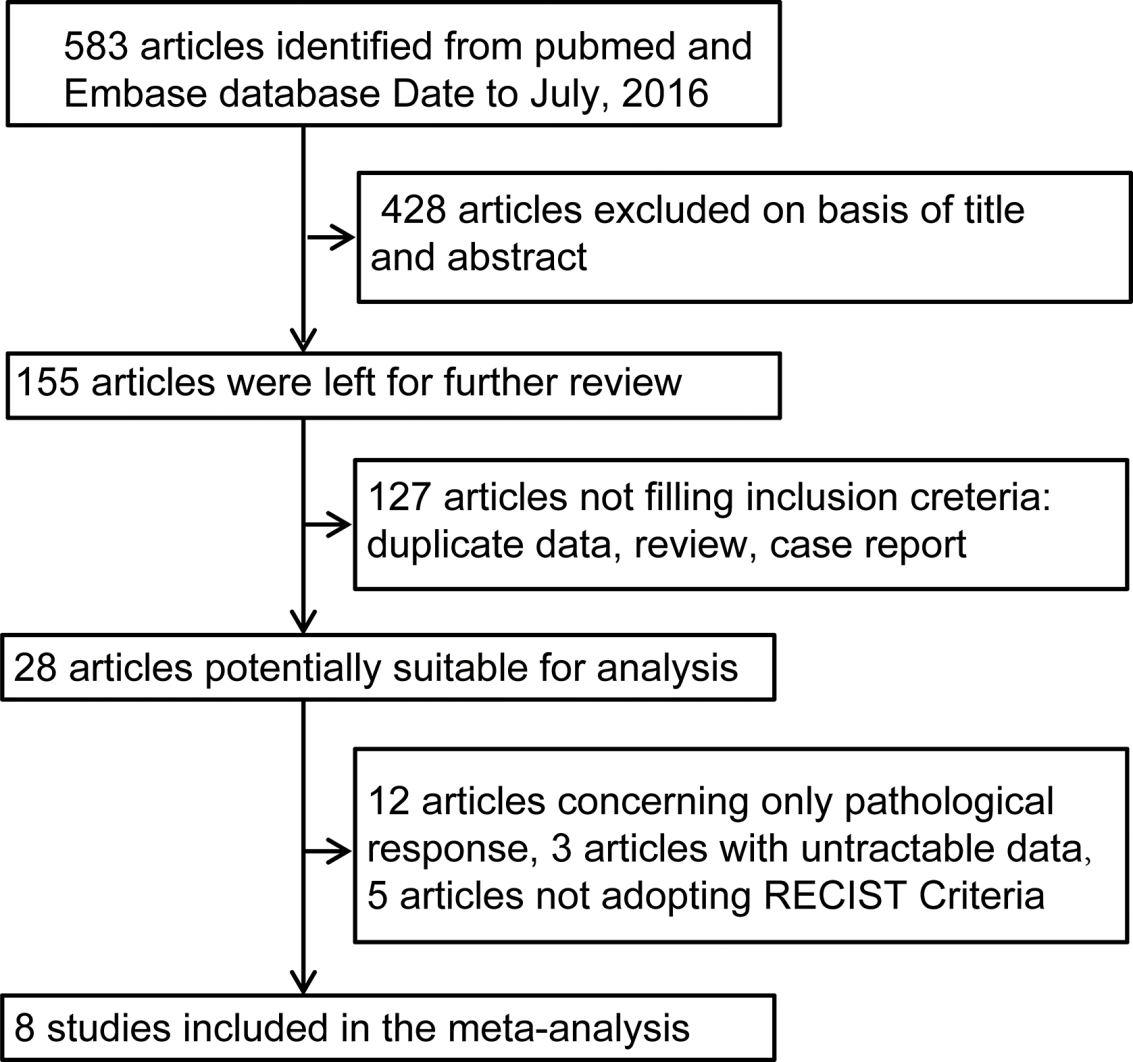
Flow chart of the meta-analysis RECIST indicates Response Evaluation Criteria in Solid Tumor.

We identified 583 unique citations using the terms described in the materials and methods. During the first round of screening, 428 citations were excluded after reading the titles and abstracts, leaving 155 articles for further assessment. During the second round of screening, 127 articles were excluded from further analysis for the reasons in Figure 1. Another 12 articles were excluded because they evaluated the pathological response but not the clinical response. The eight remaining articles were included in the pooled analysis.

### Characteristics of the studies

Table 1 shows the characteristics of the included studies, all of which reported clinical response and OS. Three articles were excluded from further analysis because they did not include detailed HR or survival curves of CR/PR vs. PD/SD (CR, Clinical Response; PR, Partial Response; PD, Progress Disease; SD, Stable Disease). Five articles were excluded because they did not use Response Evaluation Criteria in Solid Tumor (RECIST) criteria in evaluating the clinical response. The eight included studies consisted of 825 patients with 596 responders and 229 non-responders. Six studies came from Asian countries, and two came from European countries.

**Table 1:** Characteristics of studies included in the meta-analysis

Study	Country	Study period	No. of cases (non-responders)	No. of all patients	Adjustment	Follow-up period
Xie 2016 [17]	China	2003–2008	18	52	Tumor size, the expression of ALDH1	3–123 months
Fuso 2005 [29]	Italy	1997–2004	26	73	Na.	3–140 months
Liu 2013 [18]	China	2002–2011	40	103	Na.	6–113 months
Martinelli 2015 [31]	Italy	1990–2011	60	275	Pathologic response, Vaginal involvement, Lymph nodes positivity	4–119 months
Yang 2015 [30]	China	2007–2012	33	115	Na.	6–75 months
Mori 2010 [32]	Japan	2002–2006	4	30	Na.	26–83 months
Li 2012 [33]	China	2000–2011	43	154	Na.	6–142 months
Shoji 2013 [34]	Japan	2002–2011	5	23	Na.	9–90 months

### Forest plot of HR for 1-year OS

**Figure 2:**
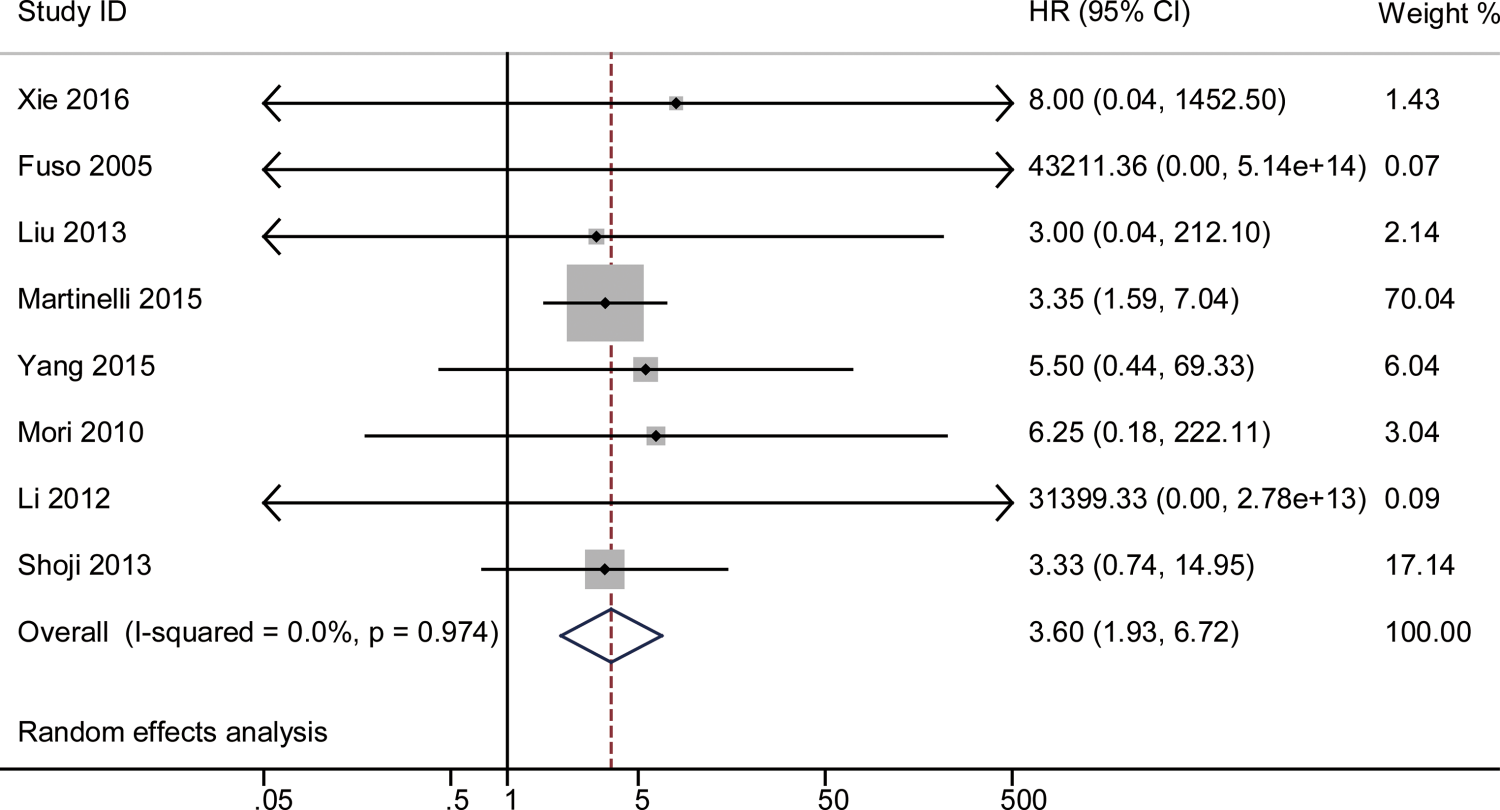
The pooled HR of non-responders vs. responders for 1-year OS The summary estimates were obtained by using a random-effects model. The data markers indicate the HRs comparing non-responders with responders among cervical cancer patients undergoing neoadjuvant chemotherapy. The size of the data markers indicates the weight of the study, which is the inverse variance of the effect estimate. The diamond data markers indicate the pooled HRs.

The HR for 1-year OS was significantly correlated with response using the random-effects model, as shown in Figure 2. The pooled HR was 3.60 for non-responders with 95% CI 1.93–6.72, compared with responders. No significant heterogeneity was observed across the studies (*P* = 0.97, *I*^2^ = 0). The same pooled HR with 95% CI 1.93–6.72 was detected in the fixed-effect model.

### Publication bias for 1-year HR: funnel plot, egger's test, and begg's test

**Figure 3:**
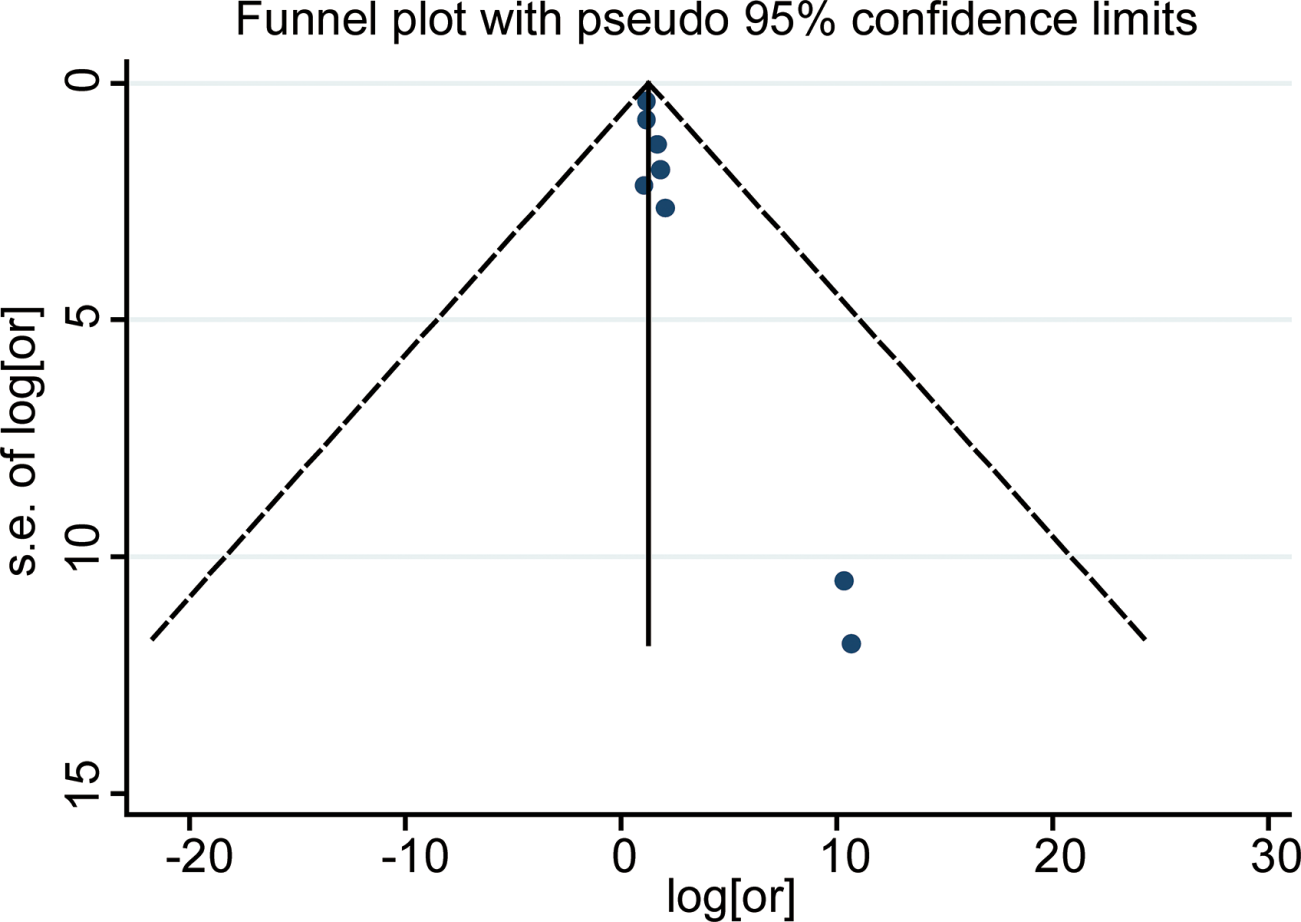
Funnel plots for detection of publication bias of HR for 1-year OS The pseudo 95% CI is computed as part of the analysis that produces the funnel plot, and corresponding to the expected 95% CI for a given standard error (SE).

A funnel plot was made for visual screening of any potential publication bias (Figure 3). Egger's test showed no obvious publication bias with *P* = 0.009 (Supplementary Figure 1). Begg's test showed a similar result with *P* = 0.06 (Continuity corrected, Supplementary Figure 2).

### Sensitivity analysis for 1-year HR

**Figure 4:**
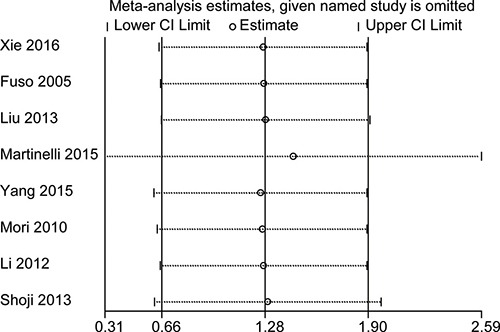
Sensitivity analysis for testing the robust of HR for 1-year OS The small circle indicates the estimated logHR, given the named study is omitted. Accordingly, the bar corresponds to the lower limit of 95% CI of the logHR.

Sensitivity analysis was performed to detect potential heterogeneity and to identify the origin of any heterogeneity. Individual studies were excluded in turn, and the results of the remaining studies were pooled. The pooled HRs slightly differed with each other, but they did not change much across the sensitivity analysis. Figure 4 illustrates that the logHRs were distributed across a range from 0.31 to 2.59. These findings mean that the HR was significantly larger than in reference 1, and that no individual study will influence the final pooled HR.

### HR for 3-year OS

**Figure 5:**
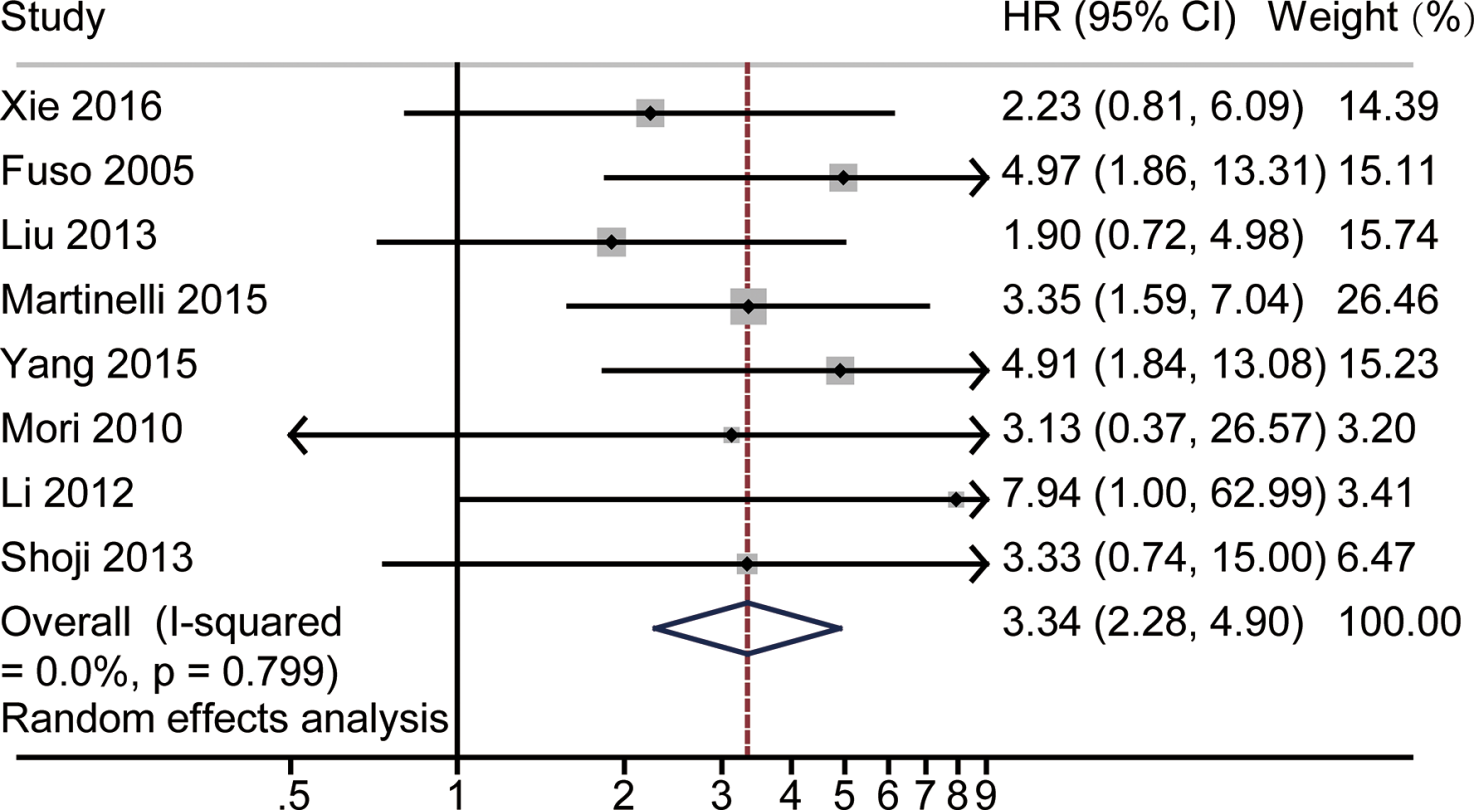
The pooled HR of non-responders vs. responders for 3-year OS The summary estimates were obtained by using a random-effects model. The data markers indicate the HRs comparing non-responder with responder among cervical cancer patients undergoing NACT. The size of the data markers indicates the weight of the study, which is the inverse variance of the effect estimate. The diamond data markers indicate the pooled HRs.

Forest plot showed that the HR for 3-year OS was significantly correlated with responsiveness, as shown in Figure 5. The pooled HR was 3.34 with 95% CI 2.28–4.90, and no significant heterogeneity was found (*P* = 0.80, *I*^2^ = 0). The random effect model was used in this analysis. A funnel plot was made for visual screening of any potential publication bias (Supplementary Figure 3). No evidence of obvious publication bias was observed by Egger's test *P* = 0.59 (Supplementary Figure 4) or Begg's test *P* = 1.00 (Supplementary Figure 5). Sensitivity analysis was performed to identify the origin of the heterogeneity. After excluding each study in turn and pooling the results of the remaining studies, no obvious variation of the HR was observed, and no heterogeneity from the included studies was identified. Supplementary Figure 6 illustrates that the logHRs were distributed across a range from 0.72 to 1.73.

### HR for 5-year OS

**Figure 6:**
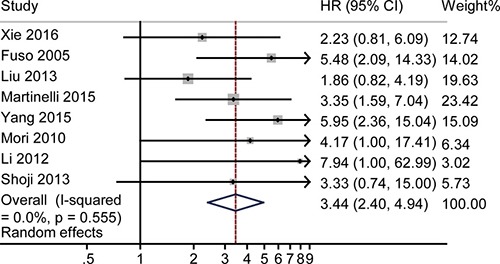
The pooled HR of non-responders vs. responders for 5-year OS The summary estimates were obtained by using a random-effects model. The data markers indicate the HRs comparing non-responders with responders among cervical cancer patients undergoing NACT. The size of the data markers indicates the weight of the study, which is the inverse variance of the effect estimate. The diamond data markers indicate the pooled HRs.

A forest plot was made to illustrate the pooled HR for 5-year OS. The fixed-effect model was used in the analysis (Figure 6). The 5-year OS rate was 3.44 (95% CI 2.40–4.94), and no significant heterogeneity was observed (*P* = 0.56, *I*^2^ = 0). Funnel plot (Supplementary Figure 7), Egger's test (Supplementary Figure 8), and Begg's test (Supplementary Figure 9) revealed no obvious publication bias (P = 0.38 for Egger's test; *P* = 0.71 for Begg's test) as shown in Supplementary Figures 8 and 9. Sensitivity analysis revealed no heterogeneity. Supplementary Figure 10 illustrates that the logHRs were distributed across a range from 0.75 to 1.79.

## DISCUSSION

In this study, we made a pooled analysis for the predictive role of NACT treatment response by combining the findings of previously published studies. Our study revealed that OS favored responders with non-responders having a relatively higher risk of mortality. Similar results were observed in 3-year and 5-year OS.

Statistical analysis showed that the present study was properly designed. The studies included in the final analysis were of satisfactory origin, and no heterogeneity was observed when pooling the data. Good balance was also observed across the studies, and no obvious bias was detected while pooling the HR. By combing the previously reported results, both the total analysis and subgroup analysis for 3-year and 5-year revealed a moderate pooling result. Sensitivity analysis showed that the result was robust, as no heterogeneity from the included studies was observed across our research.

Our result validates previous findings reported by other scholars. Li and colleagues reported a similar result: response is a significant prognostic factor for disease-free survival [15]. Xiong and colleagues also reported response to NACT was the only factor associated with survival [16]. Cai and colleagues, who performed a prospective randomized study in Central China, found that survival time of responders was significantly higher than that of non-responders (*P* = 0.0049) [11]. Professor Wu and colleagues also conducted a prospective randomized study and demonstrated that NACT response was also an independent prognostic predictor (*P* = 0.005). Our result was different from some other studies [17, 18], possibly due to sample size.

There are some disadvantages to our study. First, responsiveness by WHO criteria, which were used in many studies [10, 16, 19–21], was not evaluated in our study. Second, the role of post-surgery chemotherapy was not evaluated in the present study. In a future study, we will evaluate the prognostic role of clinical response on survival using WHO criteria. Meanwhile, whether clinical response is able to suggest the use of post-chemotherapy still need to be precisely demonstrated through large data or meta-analysis.

The role of early response (including pathological response) on long-term survival has been evaluated for quite a few solid tumors. These researches will highlight the treatment and the outcome's predicting in the future. Gadducci and colleagues also reported a new method to evaluate the early response, and they observed that patients achieving pathological response have significantly higher survival rate than others [22]. Valentini and colleagues demonstrated that early response to chemoradiation was a significant prognostic factor for patients with rectal cancer [23]. Monique and colleagues found that early response was an indicator of long-term survival indicator [24]. With increasing emphasis on early response of rectal cancer, treatment after NACT has become a topic of interest, and it has been proposed that patients with a complete early response should not undergo surgery [25].

In our systematic review and meta-analysis, we found that early response has a significant effect on survival, and it is a significant predictor of the OS rate using RECIST criteria. This finding may be useful in predicting the prognosis of cervical cancer patients and may provide some clues for their treatment.

## MATERIALS AND METHODS

### Search strategy

A literature search was first performed in December 2015 and then updated on July 1st, 2016. Medline, Pubmed, and EMBASE were searched, according to the methods introduced by the previous studies [26]. The key words were used in term of “Neoadjuvant chemotherapy” or “NACT” or “preoperative chemotherapy”, plus “response” or “responding” or “responsiveness” or “responder” or “remission”, plus “uterine cervical cancer” or “uterine cervical carcinoma” or “uterine cervical neoplasms”. The relevant references in the retrieved articles were reviewed and hand-checked to include as many eligible articles as possible.

### Study identification

Inclusion criteria: Original research articles published in a peer-reviewed journal. English was used throughout the article. Patients with definitely diagnosed cervical cancer. RECIST was used in the evaluation of clinical response. The titles, abstracts, and the articles were screened by two authors, independently to determine the eligibility.

Exclusion criteria. A total of 583 articles were searched. Then, 428 articles were excluded from analysis because of irrelevance judged by reading the abstract and title. Another 12 articles were excluded from further analysis because they did not evaluate clinical response but only pathological response. Three studies were excluded because the data of CR/PR versus PD/SD cannot be extracted [3, 27, 28]. Finally eight studies were included in the final analysis [17, 18, 29–34].

### Data extraction and quality assessment

Data extraction was independently carried out by two reviewers. The following information were extracted from the included studies: (1) the name of the first author, (2) the publication year, (3) countries and origin of the study, (4) number of cases, (5) number of controls, (6) the period of follow-up, (7) HR and 95% confidence interval (95% CI) in Cox analysis, and (8) survival curve using Kaplan-Meier method. Discrepancies were resolved by discussing with a third author or consulting with the senior investigators.

Quality assessment of the included studies was carried out by using the Newcastle-Ottawa Scale (NOS), as used in the previous studies [35, 36]. The NOS is a nine-point scale that integrates the selection process of the study (≤ 4 points), the comparability of the studies (≤ 2 points), and the identification of the exposure and the outcome of the study participants (≤ 3 points). Two reviewers independently performed quality assessment as well as the risk of bias. Selection bias, statistical rationality, measurement error, and representativeness for the included studies were also studied. Disagreements were resolved by a third reviewer or consulting with the senior investigators.

### Statistical analyses

The HR was used as the common measure of association across the studies. Meanwhile, forest plots were employed to visually illustrate the HR and corresponding 95% CI across the studies. Cochrane *Q* statistic was used to test the heterogeneity across the studies, and the significance level was set at *P* < 0.10 [26]. Meanwhile, *I*^2^ statistic was employed to test the heterogeneity across the studies, and it was considered to be significant if *I*^2^ > 50% [35]. The pooled HR was first calculated by using random effect model based on the DerSimonian and Laird method [37]. To inspect the differences between the random effect model and the fixed-effect model, we further calculated the pooled HR by using the fixed-effect model based on the Mantel-Haenszel method [38]. The possibility of publication bias was assessed by visually screening a funnel plot. Begg's test and Egger's test were also used to assess the publication bias [39–41]. Sensitivity analysis was carried out to assess the robustness of the study [26], and one study was omitted from each analysis to test the robustness of the combined results. Stata version 11 (StataCorp, College Station, TX, USA) was used for the statistical analysis. Difference with two-sided *P* < 0.05 was considered to be statistically significant.

When data including HR and 95% CI could not be obtained directly from the articles, Engauge Digitizer 4.1 were used to read the survival curve of the included studies [42], based on the calculus theory and integral theory [43, 44]. Engauge Digitizer was provided as a free software by previous scholars, and can be freely downloaded from http://digitizer.sourceforge.net, with instructions for readers [45].

Subgroup analysis was also performed based on the follow-up period; in this research, we calculated the pooled HR of 3-year survival as well as pooled HR of 5-year survival. The above software were also employed during the subgroup statistical analysis.

## SUPPLEMENTARY MATERIALS FIGURES AND TABLES



## References

[1] Torre LA, Bray F, Siegel RL, Ferlay J, Lortet-Tieulent J, Jemal A (2015). Global cancer statistics, 2012. CA Cancer J Clin.

[2] Chen W, Zheng R, Baade PD, Zhang S, Zeng H, Bray F, Jemal A, Yu XQ, He J (2016). Cancer statistics in China, 2015. CA Cancer J Clin.

[3] Shoji T, Takatori E, Furutake Y, Takada A, Nagasawa T, Omi H, Kagabu M, Honda T, Miura F, Takeuchi S, Kumagai S, Yoshizaki A, Sato A (2016). Phase II clinical study of neoadjuvant chemotherapy with CDDP/CPT-11 regimen in combination with radical hysterectomy for cervical cancer with a bulky mass. Int J Clin Oncol.

[4] Takatori E, Shoji T, Omi H, Kagabu M, Miura F, Takeuchi S, Kumagai S, Yoshizaki A, Sato A, Sugiyama T (2015). Analysis of prognostic factors for patients with bulky squamous cell carcinoma of the uterine cervix who underwent neoadjuvant chemotherapy followed by radical hysterectomy. Int J Clin Oncol.

[5] Liang Y, Lu B, Chen X, Qin J, Cheng X, Xie X, Lu W (2016). Prognostic value of pathological response to neoadjuvant chemotherapy in bulky stage Ib2 and IIa cervical squamous cell cancer patients. Virchows Arch.

[6] Cheng XD, Lu WG, Ye F, Wan XY, Xie X (2009). The association of XRCC1 gene single nucleotide polymorphisms with response to neoadjuvant chemotherapy in locally advanced cervical carcinoma. J Exp Clin Cancer Res.

[7] Sardi JE, di Paola GR, Giaroli A, Sananes C, Gomez Rueda N, Cachau A, Vighi S, Burlando S (1988). Results of a phase II trial with neoadjuvant chemotherapy in carcinoma of the cervix uteri. Gynecol Oncol.

[8] Sardi JE, Giaroli A, Sananes C, Ferreira M, Soderini A, Bermudez A, Snaidas L, Vighi S, Gomez Rueda N, di Paola G (1997). Long-term follow-up of the first randomized trial using neoadjuvant chemotherapy in stage Ib squamous carcinoma of the cervix: the final results. Gynecol Oncol.

[9] Sardi JE, Boixadera MA, Sardi JJ (2005). Neoadjuvant chemotherapy in cervical cancer: a new trend. Curr Opin Obstet Gynecol.

[10] Hu T, Li S, Chen Y, Shen J, Li X, Huang K, Yang R, Wu L, Chen Z, Jia Y, Wang S, Cheng X, Han X (2012). Matched-case comparison of neoadjuvant chemotherapy in patients with FIGO stage IB1-IIB cervical cancer to establish selection criteria. Eur J Cancer.

[11] Cai HB, Chen HZ, Yin HH (2006). Randomized study of preoperative chemotherapy versus primary surgery for stage IB cervical cancer. J Obstet Gynaecol Res.

[12] Bentivegna E, Gouy S, Maulard A, Chargari C, Leary A, Morice P (2016). Oncological outcomes after fertility-sparing surgery for cervical cancer: a systematic review. Lancet Oncol.

[13] Rob L, Skapa P, Robova H (2011). Fertility-sparing surgery in patients with cervical cancer. Lancet Oncol.

[14] Morice P, Uzan C, Gouy S, Verschraegen C, Haie-Meder C (2012). Gynaecological cancers in pregnancy. Lancet.

[15] Li X, Huang K, Zhang Q, Shen J, Zhou H, Yang R, Wang L, Liu J, Zhang J, Sun H, Jia Y, Du X, Wang H (2016). Early response to neoadjuvant chemotherapy can help predict long-term survival in patients with cervical cancer. Oncotarget.

[16] Xiong Y, Liang LZ, Cao LP, Min Z, Liu JH (2011). Clinical effects of irinotecan hydrochloride in combination with cisplatin as neoadjuvant chemotherapy in locally advanced cervical cancer. Gynecol Oncol.

[17] Xie Q, Liang J, Rao Q, Xie X, Li R, Liu Y, Zhou H, Han J, Yao T, Lin Z (2016). Aldehyde Dehydrogenase 1 Expression Predicts Chemoresistance and Poor Clinical Outcomes in Patients with Locally Advanced Cervical Cancer Treated with Neoadjuvant Chemotherapy Prior to Radical Hysterectomy. Ann Surg Oncol.

[18] Liu SP, Yang JX, Cao DY, Shen K, Xiang Y, Lang JH (2014). Efficacy of neoadjuvant cisplatin and 5-flourouracil prior to surgery in FIGO stage IB2/IIA2 cervical cancer. Mol Clin Oncol.

[19] Robova H, Rob L, Halaska MJ, Pluta M, Skapa P, Strnad P, Lisy J, Komar M (2013). High-dose density neoadjuvant chemotherapy in bulky IB cervical cancer. Gynecol Oncol.

[20] Selvaggi L, Loizzi V, Di Gilio AR, Nardelli C, Cantatore C, Cormio G (2006). Neoadjuvant chemotherapy in cervical cancer: a 67 patients experience. Int J Gynecol Cancer.

[21] MacLeod C, O’Donnell A, Tattersall MH, Dalrymple C, Firth I (2001). Locally advanced cervix cancer: chemotherapy prior to definitive surgery or radiotherapy. A single institutional experience. Australas Radiol.

[22] Gadducci A, Sartori E, Maggino T, Zola P, Cosio S, Zizioli V, Lapresa M, Piovano E, Landoni F (2013). Pathological response on surgical samples is an independent prognostic variable for patients with Stage Ib2-IIb cervical cancer treated with neoadjuvant chemotherapy and radical hysterectomy: an Italian multicenter retrospective study (CTF Study). Gynecol Oncol.

[23] Valentini V, Coco C, Picciocchi A, Morganti AG, Trodella L, Ciabattoni A, Cellini F, Barbaro B, Cogliandolo S, Nuzzo G, Doglietto GB, Ambesi-Impiombato F, Cosimelli M (2002). Does downstaging predict improved outcome after preoperative chemoradiation for extraperitoneal locally advanced rectal cancer? A long-term analysis of 165 patients. Int J Radiat Oncol Biol Phys.

[24] Maas M, Nelemans PJ, Valentini V, Das P, Rodel C, Kuo LJ, Calvo FA, Garcia-Aguilar J, Glynne-Jones R, Haustermans K, Mohiuddin M, Pucciarelli S, Small W (2010). Long-term outcome in patients with a pathological complete response after chemoradiation for rectal cancer: a pooled analysis of individual patient data. Lancet Oncol.

[25] Appelt AL, Ploen J, Harling H, Jensen FS, Jensen LH, Jorgensen JC, Lindebjerg J, Rafaelsen SR, Jakobsen A (2015). High-dose chemoradiotherapy and watchful waiting for distal rectal cancer: a prospective observational study. Lancet Oncol.

[26] Pan A, Wang Y, Talaei M, Hu FB (2015). Relation of Smoking With Total Mortality and Cardiovascular Events Among Patients With Diabetes Mellitus: A Meta-Analysis and Systematic Review. Circulation.

[27] Sebastiao AM, da Silva Rocha LS, Gimenez RD, de Barros LA, Fukushima JT, da Silva SC, da Costa Miranda V, de Souza Caires IQx, de Freitas D, Filho EA, Diz Mdel P (2016). Carboplatin-based chemoradiotherapy in advanced cervical cancer: an alternative to cisplatin-based regimen?. Eur J Obstet Gynecol Reprod Biol.

[28] Park DC, Suh MJ, Yeo SG (2009). Neoadjuvant paclitaxel and cisplatin in uterine cervical cancer: long-term results. Int J Gynecol Cancer.

[29] Fuso L, Mazzola S, Marocco F, Ferrero A, Dompe D, Carus AP, Zola P (2005). Pretreatment serum hemoglobin level as a predictive factor of response to neoadjuvant chemotherapy in patients with locally advanced squamous cervical carcinoma: a preliminary report. Gynecol Oncol.

[30] Yang L, Guo J, Shen Y, Cai J, Xiong Z, Dong W, Min J, Wang Z (2015). Clinical efficacy and safety of paclitaxel plus carboplatin as neoadjuvant chemotherapy prior to radical hysterectomy and pelvic lymphadenectomy for Stage IB2–IIB cervical cancer. Int J Clin Exp Med.

[31] Martinelli F, Bogani G, Ditto A, Carcangiu M, Papadia A, Lecce F, Chiappa V, Lorusso D, Raspagliesi F (2015). How often parametrial involvement leads to post-operative adjuvant treatment in locally advanced cervical cancer after neoadjuvant chemotherapy and type C radical hysterectomy?. Eur J Surg Oncol.

[32] Mori T, Hosokawa K, Sawada M, Kuroboshi H, Tatsumi H, Koshiba H, Okubo T, Kitawaki J (2010). Neoadjuvant weekly carboplatin and paclitaxel followed by radical hysterectomy for locally advanced cervical cancer: long-term results. Int J Gynecol Cancer.

[33] Li R, Lu ST, Si JG, Liu B, Wang H, Mei YY, Linghu H (2013). Prognostic value of responsiveness of neoadjuvant chemotherapy before surgery for patients with stage IB(2)/IIA(2) cervical cancer. Gynecol Oncol.

[34] Shoji T, Takatori E, Saito T, Omi H, Kagabu M, Miura F, Takeuchi S, Sugiyama T (2013). Neoadjuvant chemotherapy using platinum- and taxane-based regimens for bulky stage Ib2 to IIb non-squamous cell carcinoma of the uterine cervix. Cancer Chemother Pharmacol.

[35] Bi H, Gan Y, Yang C, Chen Y, Tong X, Lu Z (2015). Breakfast skipping and the risk of type 2 diabetes: a meta-analysis of observational studies. Public Health Nutr.

[36] Stang A (2010). Critical evaluation of the Newcastle-Ottawa scale for the assessment of the quality of nonrandomized studies in meta-analyses. Eur J Epidemiol.

[37] DerSimonian R, Laird N (1986). Meta-analysis in clinical trials. Control Clin Trials.

[38] Tobias DK, Pan A, Jackson CL, O’Reilly EJ, Ding EL, Willett WC, Manson JE, Hu FB (2014). Body-mass index and mortality among adults with incident type 2 diabetes. N Engl J Med.

[39] Begg CB, Mazumdar M (1994). Operating characteristics of a rank correlation test for publication bias. Biometrics.

[40] Egger M, Davey Smith G, Schneider M, Minder C (1997). Bias in meta-analysis detected by a simple, graphical test. BMJ.

[41] Zhu B, Sun Y, Qi L, Zhong R, Miao X (2015). Dietary legume consumption reduces risk of colorectal cancer: evidence from a meta-analysis of cohort studies. Sci Rep.

[42] Wu XL, Tu Q, Faure G, Gallet P, Kohler C, Bittencourt Mde C (2016). Diagnostic and Prognostic Value of Circulating Tumor Cells in Head and Neck Squamous Cell Carcinoma: a systematic review and meta-analysis. Sci Rep.

[43] Guyot P, Ades AE, Ouwens MJ, Welton NJ (2012). Enhanced secondary analysis of survival data: reconstructing the data from published Kaplan-Meier survival curves. BMC Med Res Methodol.

[44] Williamson PR, Smith CT, Hutton JL, Marson AG (2002). Aggregate data meta-analysis with time-to-event outcomes. Stat Med.

[45] Tierney JF, Stewart LA, Ghersi D, Burdett S, Sydes MR (2007). Practical methods for incorporating summary time-to-event data into meta-analysis. Trials.

