# Supplementary material for: Meta-analysis showing that early response to neoadjuvant chemotherapy predicts better survival among cervical cancer patients
Source: Oncotarget. 2017 Jul 21;8(35):59609–17. doi: 10.18632/oncotarget.19425 (PMC5601760; doi:10.18632/oncotarget.19425)
Supplement: Supplementary file 1 [file oncotarget-08-59609-s001.pdf]

Meta-analysis showing that early response to neoadjuvant chemotherapy predicts better survival among cervical cancer patients

SUPPLEMENTARY MATERIALS

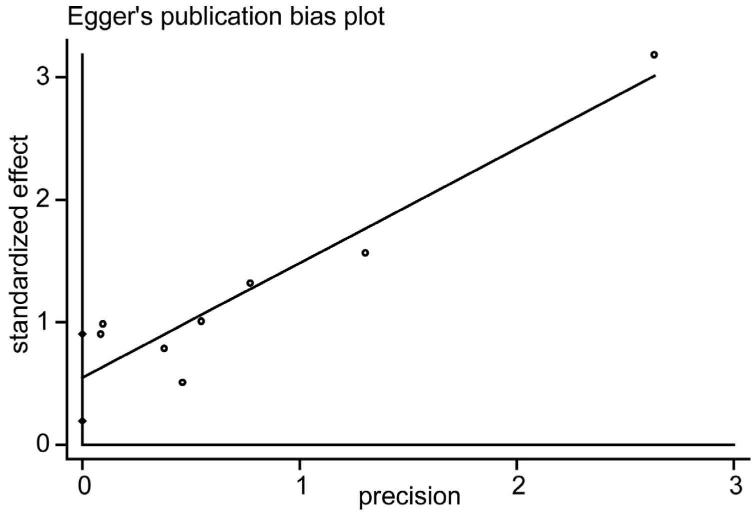

Supplementary Figure 1: Egger's publication bias plot for HR of 1-year OS. Legend: Publication bias can be concluded by intercept as well as *p*-value. The test showed that no obvious publication bias was observed with $P = 0.98$ and intercept near zero.

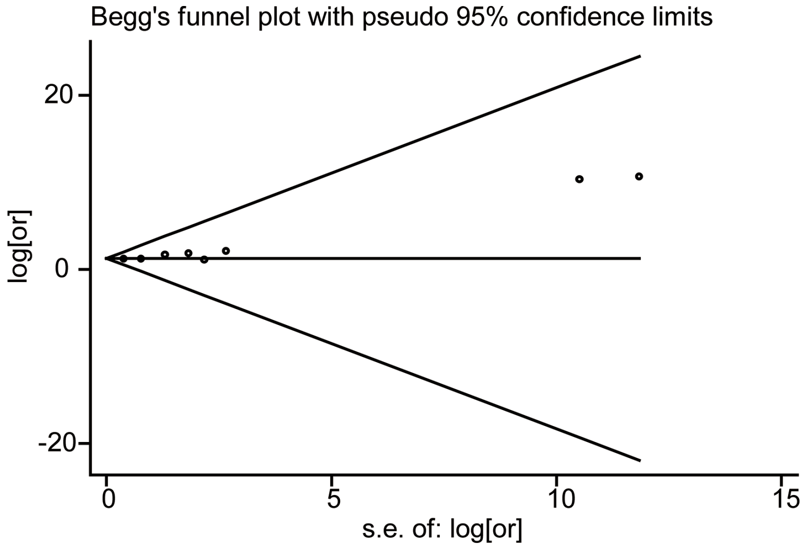

Supplementary Figure 2: Begg's funnel plot of publication bias for HR of 1-year OS. Legend: The pseudo 95% confidence interval (CI) is computed as part of the analysis that produces the funnel plot, and corresponding to the expected 95% CI for a given standard error (SE). HR indicates hazard ratio.

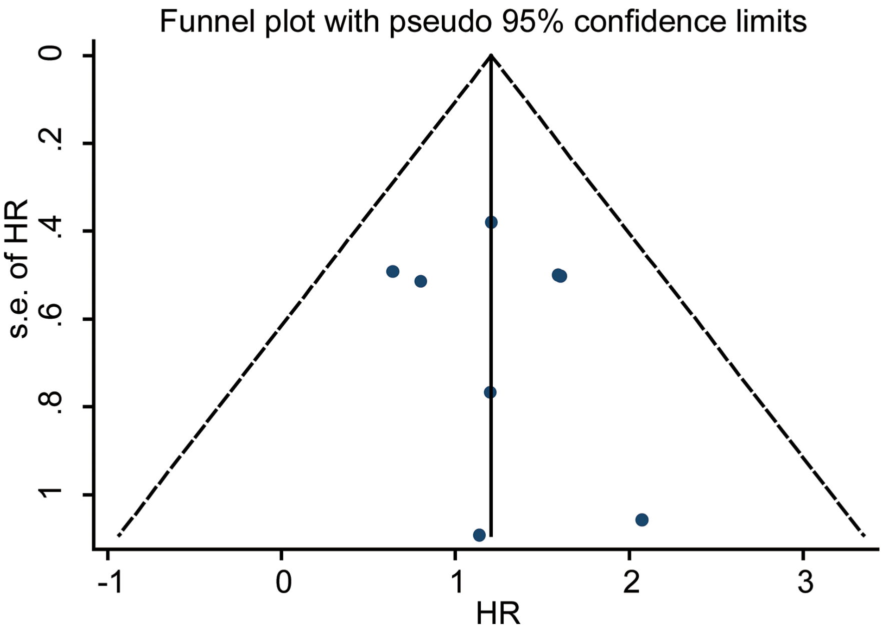

Supplementary Figure 3: Funnel plots for detection of publication bias for HR of 3-year OS. Legend: The pseudo 95% confidence interval (CI) is computed as part of the analysis that produces the funnel plot, and corresponding to the expected 95% CI for a given standard error (SE). HR indicates hazard ratio.

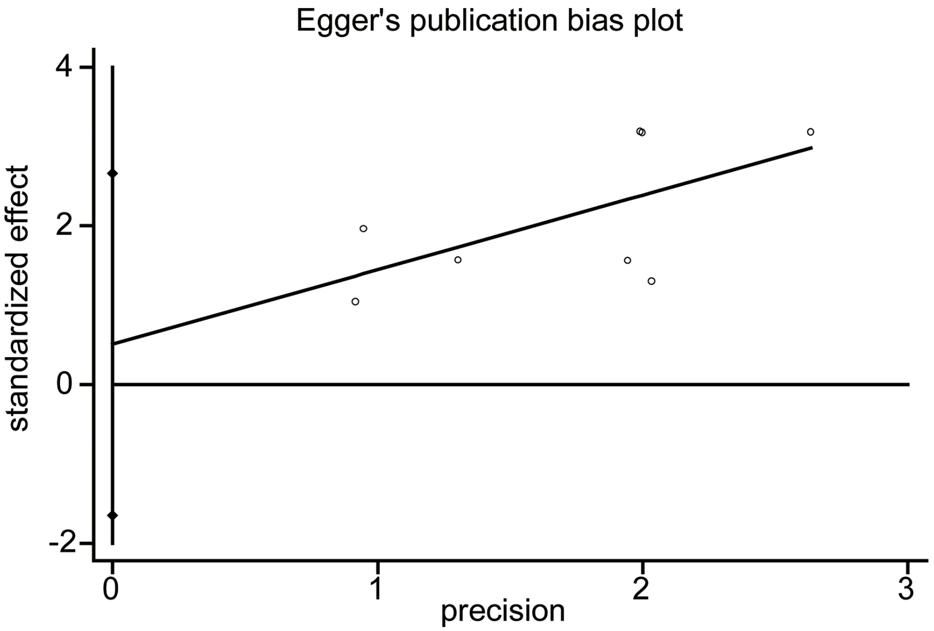

Supplementary Figure 4: Egger's publication bias plot for HR of 3-year OS. Legend: Publication bias can be concluded by intercept as well as p-value. The test showed that no obvious publication bias was observed with $P = 0.59$ and intercept near zero.

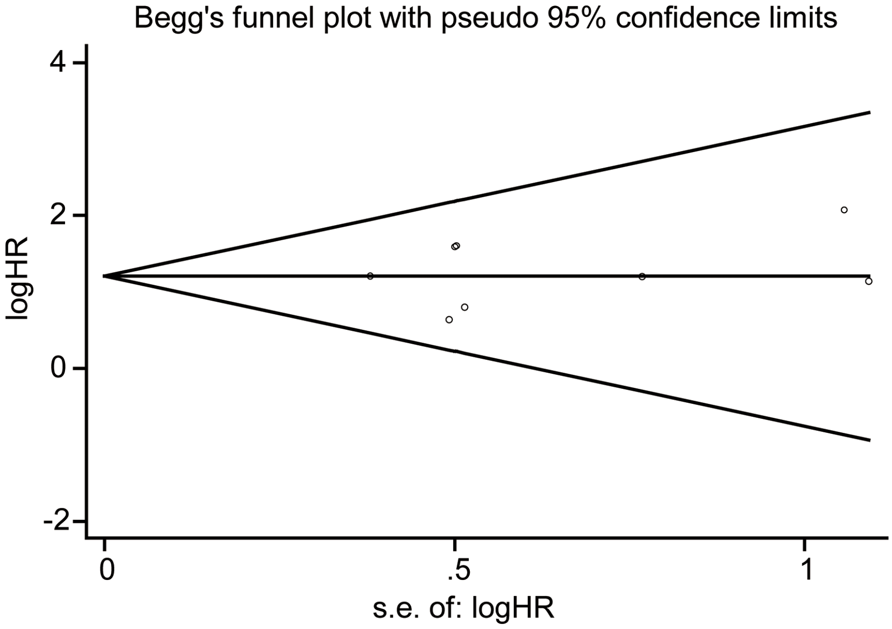

Supplementary Figure 5: Begg's funnel plot of publication bias for HR of 3-year OS. Legend: The pseudo 95% confidence interval (CI) is computed as part of the analysis that produces the funnel plot, and corresponding to the expected 95% CI for a given standard error (SE). HR indicates hazard ratio.

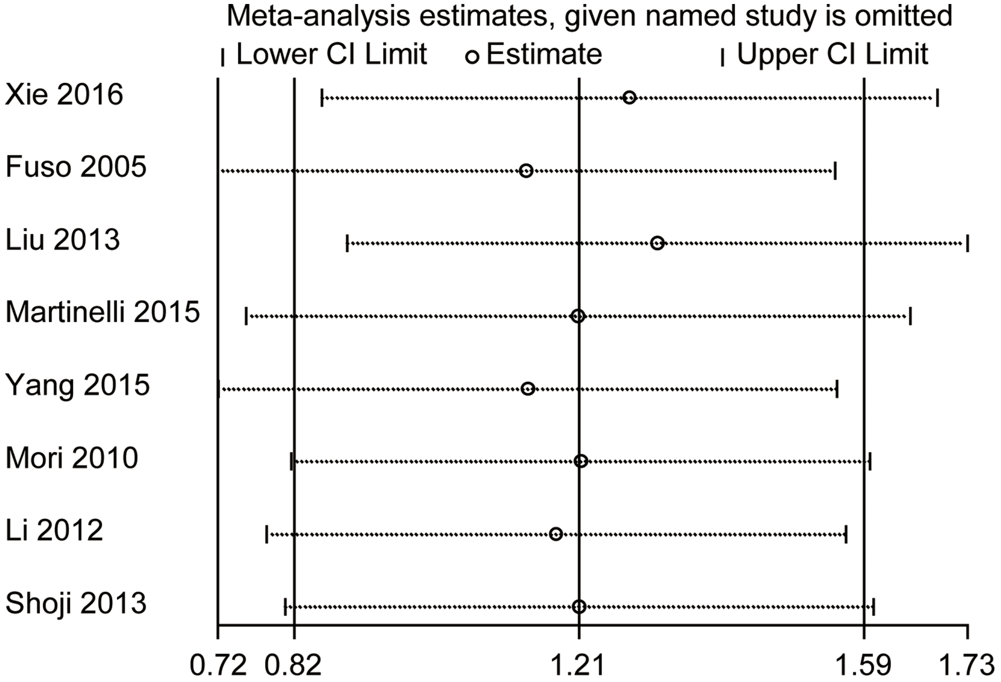

Supplementary Figure 6: Sensitivity analysis for testing the robust of the pooled HR of 3-year OS. Legend: The small circle indicates the estimated logHR, given the named study is omitted. Accordingly, the bar is corresponding to the lower limit of 95% CI of the logHR. The Figure indicates that the result is robust.

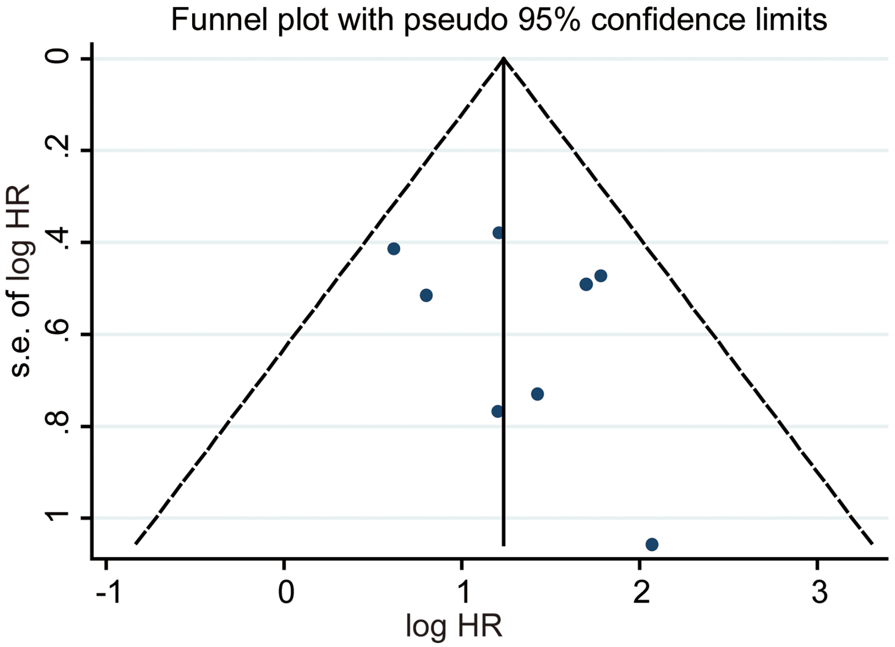

Supplementary Figure 7: Funnel plots for detection of publication bias for HR of 5-year OS. Legend: The pseudo 95% confidence interval (CI) is computed as part of the analysis that produces the funnel plot, and corresponding to the expected 95% CI for a given standard error (SE). HR indicates hazard ratio.

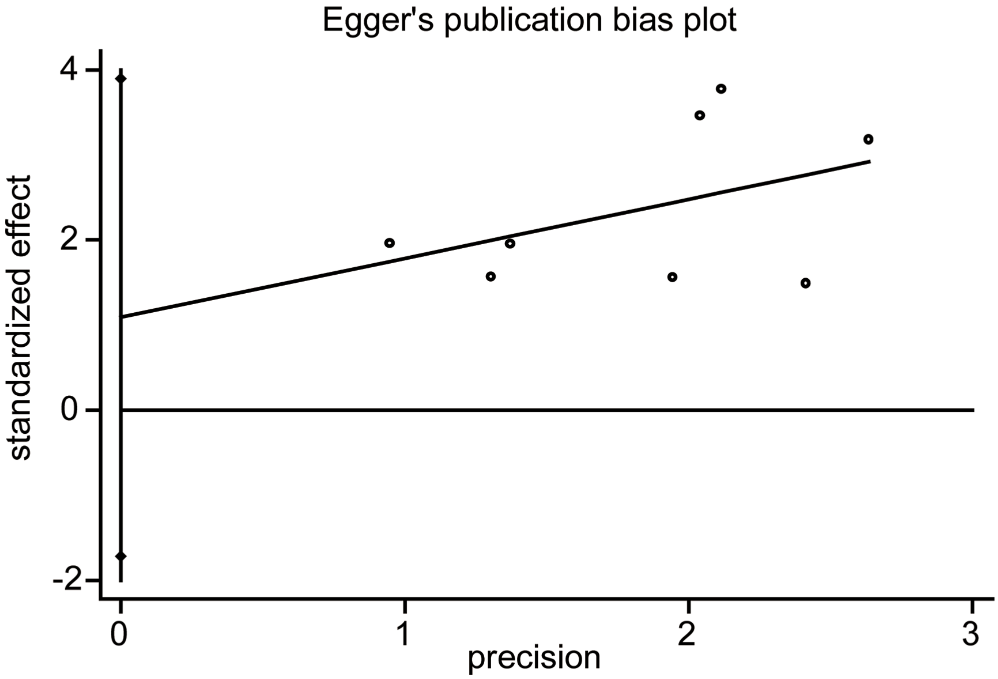

Supplementary Figure 8: Egger's publication bias plot for HR of 5-year OS. Legend: Publication bias can be concluded by intercept as well as *p*-value. The test showed that no obvious publication bias was observed with *P* = 0.38 and intercept near zero.

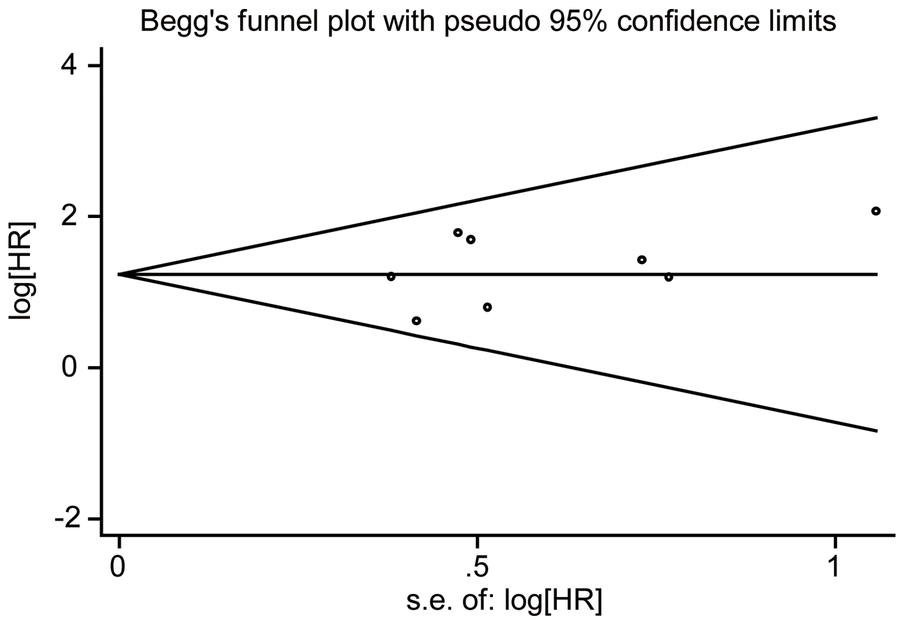

Supplementary Figure 9: Begg's funnel plot of publication bias for HR of 5-year OS. Legend: The pseudo 95% confidence interval (CI) is computed as part of the analysis that produces the funnel plot, and corresponding to the expected 95% CI for a given standard error (SE). HR indicates hazard ratio.

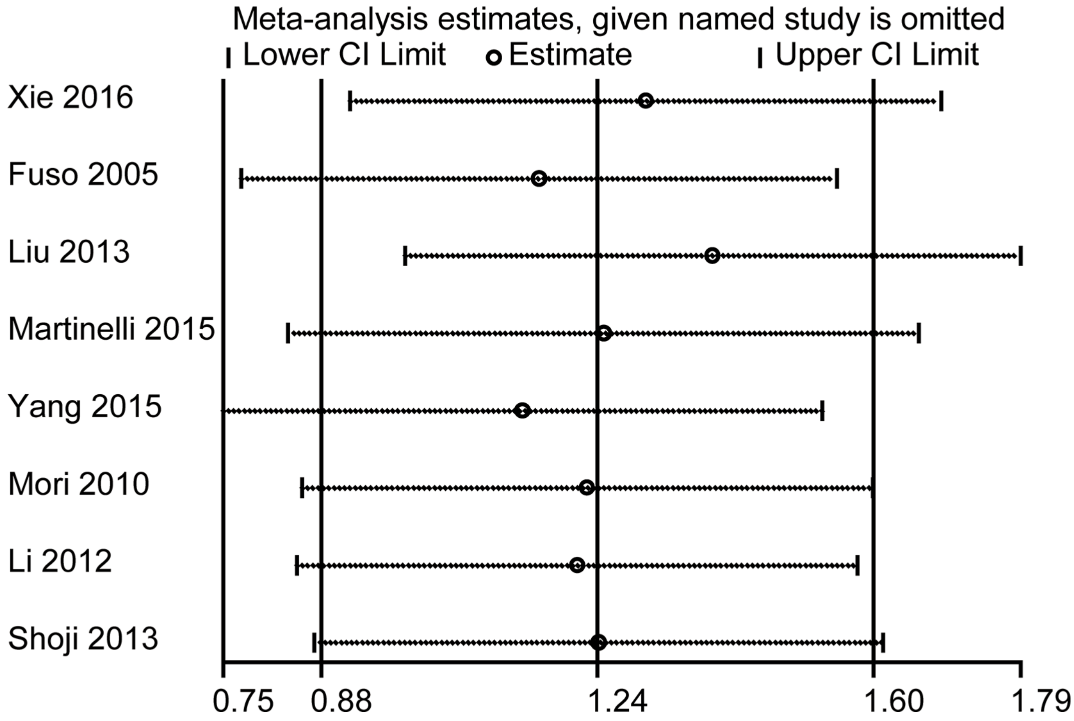

Supplementary Figure 10: Sensitivity analysis for testing the robust of the pooled HR of 5-year OS. Legend: The small circle indicates the estimated logHR, given the named study is omitted. Accordingly, the bar is corresponding to the lower limit of 95% CI of the logHR. The Figure indicates that the result is robust.
